# Polymerase Gamma Mitochondrial DNA Depletion Syndrome Initially Presenting as Disproportionate Respiratory Distress in a Moderately Premature Neonate: A Case Report

**DOI:** 10.3389/fgene.2021.664278

**Published:** 2021-06-14

**Authors:** Andrew D. Franklin, Bimal P. Chaudhari, Daniel C. Koboldt, Kerri Z. Machut

**Affiliations:** ^1^Division of Neonatology, NorthShore University HealthSystem, Evanston, IL, United States; ^2^Division of Genetic and Genomic Medicine, Nationwide Children’s Hospital, Columbus, OH, United States; ^3^Division of Neonatology, Nationwide Children’s Hospital, Columbus, OH, United States; ^4^The Steve and Cindy Rasmussen Institute for Genomic Medicine at Nationwide Children’s Hospital, Columbus, OH, United States; ^5^Department of Pediatrics, The Ohio State University College of Medicine, Columbus, OH, United States; ^6^Division of Neonatology, Ann & Robert H. Lurie Children’s Hospital of Chicago, Chicago, IL, United States; ^7^Department of Pediatrics, Northwestern University Feinberg School of Medicine, Chicago, IL, United States

**Keywords:** polymerase gamma, mitochondria DNA depletion, pulmonary hypertension, whole exome sequencing, case report

## Abstract

A 32-week premature infant presented with respiratory failure, later progressing to pulmonary hypertension (PH), liver failure, lactic acidosis, and encephalopathy. Using exome sequencing, this patient was diagnosed with a rare Polymerase Gamma (POLG)-related mitochondrial DNA (mtDNA) depletion syndrome. This case demonstrates that expanding the differential to uncommon diagnoses is important for complex infants, even in premature neonates whose condition may be explained partially by their gestational age (GA). It also shows that patients with complex neonatal diseases with significant family history may benefit from exome sequencing for diagnosis.

## Introduction

Human mitochondrial DNA (mtDNA) is circular double stranded DNA that encodes the respiratory chain proteins and associated RNA apparatus, which is involved in maintenance and translation of respiratory chain proteins. Maintenance of the mtDNA relies on transcription and translation of nuclear genes. These nuclear genes facilitate mitochondrial nucleotide synthesis or mtDNA replication. mtDNA depletion disorders (OMIM #PS603041) are a heterogeneous group of mostly autosomal recessive diseases classified by a profound reduction in mtDNA copy number from defects of either of these two mechanisms.

Depletion of mtDNA causes a variable degree of energy failure with four main clinical presentations, all of which can begin in infancy. Myopathic disease results from a defect in the mitochondrial isoform of thymidine kinase. This condition presents with feeding difficulty, failure to thrive, hypotonia, motor regression, and early death. Hepatocerebral disease results from a defect in DGUOK, Polymerase Gamma (POLG), or MPV17 and presents with persistent vomiting, failure to thrive, lactic acidosis, hypotonia, liver dysfunction, and encephalopathy. Encephalomyopathic disease results from a defect in the beta subunit of ADP-forming succinyl CoA synthase and is characterized by dystonia, hypotonia, hearing impairment, and epilepsy. Mitochondrial neurogastrointestinal encephalopathy syndrome (MNGIE) is caused by mutations in TYMP and presents with predominantly GI symptoms.

First discovered in 2001, POLG-related disorders are a subset of mtDNA depletion disorders with a defect in the catalytic subunit of the mtDNA polymerase, one of the three essential factors required for mtDNA replication. These disorders cause progressive accumulation of errors in, or depletion of, mtDNA, leading to an oxidative phosphorylation defect. The POLG gene has greater than 200 mutations distributed across three domains (polymerase, exonuclease, and linker) that lead to clinical disease. Carrier frequency of these mutations is high in the western world, ~1/100 in North America (Hakonen et al., 2007; Wong et al., 2008; Stumpf et al., 2013).

Several specific phenotypes of POLG-related diseases have been classified, although few are diagnosed in the neonatal period; seldom are they described in premature infants. Alpers-Huttenlocher syndrome is one of the most severe phenotypes with childhood-onset, progressive encephalopathy with intractable epilepsy, and liver failure. The childhood myocerebrohepatopathy spectrum has onset in the first few months to 3 years and presents with developmental delay, lactic acidosis, myopathy, and liver failure. The myoclonic epilepsy myopathy sensory ataxia spectrum presents with epilepsy, myopathy, and ataxia without ophthalmoplegia. The ataxia neuropathy spectrum presents with ataxia and neuropathy, but may develop ophthalmoplegia and seizures. Autosomal recessive progressive external ophthalmoplegias presents with weakness of the eye muscles and may develop other symptoms over decades. Autosomal dominant progressive external ophthalmoplegia, however, presents with myopathy, axonal neuropathy, ataxia, parkinsonism, cataracts, and may have hearing loss (Cohen and Naviaux, 2010; Nurminen et al., 2017). These types present in childhood, but age of onset can be variable and extend into adulthood. We present an unusual early neonatal presentation of POLG mtDNA depletion syndrome in a premature infant. This case demonstrates the importance of broad genomic testing in premature infants with atypical presentations.

## Case Report

### Maternal/Birth History

A female infant was born at 32 0/7 weeks gestational age (GA) *via* c-section for fetal bradycardia. The mother was 33 years old, gravida 4 para 3. Maternal labs revealed GBS positive status and negative serologies. The mother received peri-partum steroids for fetal lung maturity. Routine prenatal ultrasound was unremarkable other than increased density of the lateral ventricles. Family history revealed a sibling born at 31 weeks gestation with death at 6 days of life from severe respiratory failure out of proportion with GA. That infant also had generalized edema, mild transaminitis, and normal echocardiography. The underlying etiology was undetermined. Two other siblings were born full term without complications and remained healthy.

After delivery, the infant received bag-mask ventilation for apnea and bradycardia, then weaned to CPAP. Her exam initially showed hypotonia, which improved with resuscitation. Apgars were 2 at 1 min and 7 at 5 min of life. Birthweight was 1,670 g (50th percentile).

### NICU Course

**Figure 1:**
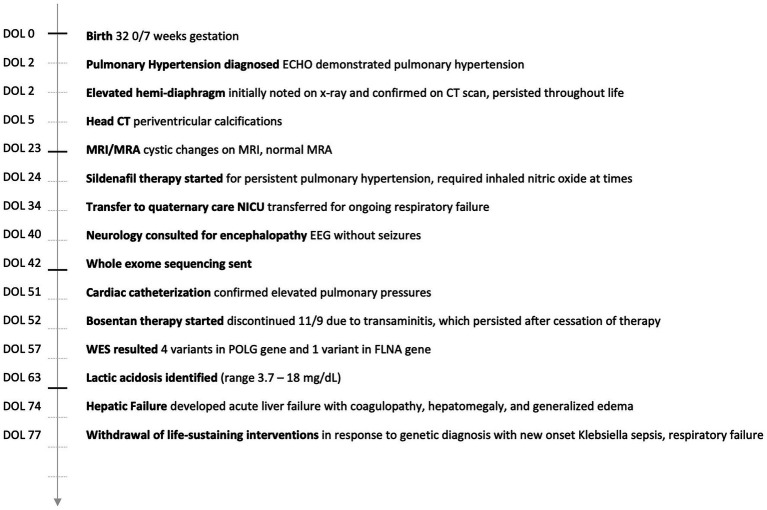
Case timeline.

She was admitted to her delivery hospital NICU and transferred to our children’s hospital at 1 month of age for ongoing respiratory failure on day of life (DOL) 34 (see Figure 1). She required mechanical ventilation shortly after birth. Initial chest radiography demonstrated signs of respiratory distress syndrome (RDS) and an elevated right hemi-diaphragm; she received three doses of surfactant. Initial echocardiography showed pulmonary hypertension (PH).

Throughout the NICU course, her respiratory failure and elevated hemi-diaphragm persisted. She was supported with CPAP and mechanical ventilation. CT chest showed bilateral ground glass appearance, a right middle lobe hyperlucency, without evidence of pulmonary hypoplasia. A next generation sequencing panel for congenital alveolar proteinosis including ABCA3, FOXF1, NKX2-1, SFTPB, and SFTPC did not reveal a diagnosis. Ultrasound of the diaphragm showed equal movement bilaterally. Bronchoscopy by otolaryngology was normal. She was treated with diuretics and systemic corticosteroids.

Echocardiography and cardiac catheterization showed persistent PH. She received inhaled nitric oxide and milrinone without clinical improvement. Subsequently, she received sildenafil and later, bosentan. Soon after, her previously normal transaminases became elevated. Despite discontinuation of bosentan, her transaminases remained elevated, and she developed acute liver failure with coagulopathy, hepatomegaly, and generalized edema around 3 months of age.

Head CT showed periventricular calcifications, likely consistent with earlier infarction and ventricular asymmetry, but cytomegalovirus, toxoplasmosis, and brain MRI/MRA were unremarkable. The infant showed increasing signs of encephalopathy with decreased activity over time. Video EEG showed no seizure activity. Ammonia, serum amino acids, acylcarnitine profile, very long chain fatty acids, and urine organic acids were normal. Her creatinine was decreased as low as 0.07 mg/dL. Lactic acid was initially normal, but at 2 months of age became persistently elevated (maximum 18 mg/dL).

**Figure 2:**
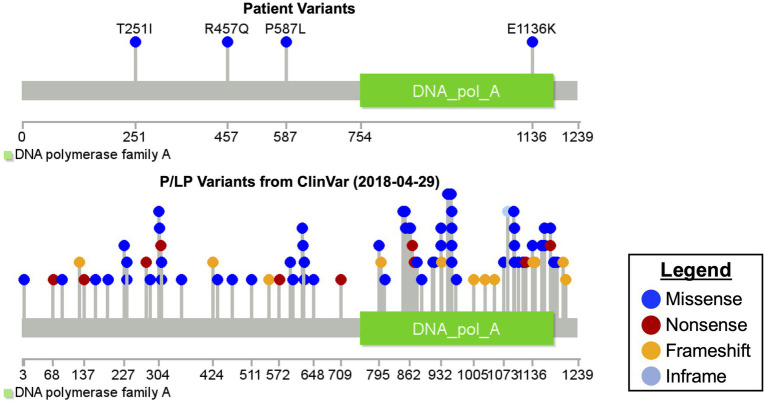
Mutation diagram of polymerase gamma.

Microarray and newborn screens were normal. Given the family history and lack of a unifying diagnosis, trio (mother, father, and proband) rapid exome sequencing (rES) was performed by send out to a commercial reference lab (GeneDx, MD, United States) and showed four variants in the POLG gene and one variant in the FLNA gene (see Table 1 for details on specific variants and classification and Figure 2 for relative location of variants). Zygosity and inheritance were verified by Sanger sequencing, and selected variants (with the exception of R457Q) were further assessed in both affected and unaffected siblings to aid in interpretation. Three *POLG* variants were present on the maternal allele. Of these, the p.T251I and p.P587L missense variants are typically found in *cis*, segregating with disease (Lamantea et al., 2002; Lamantea and Zeviani, 2004) when in *trans* with a pathogenic variant and together they account for approximately 6% of disease-causing alleles in the POLG gene (Tang et al., 2011). While these variants are listed in ClinVar (Landrum et al., 2018) as having conflicting interpretations, six out of seven assertions, which are not likely pathogenic or pathogenic are related to a phenotype of progressive sclerosis poliodystrophy (the 7th does not list a phenotype; https://www.ncbi.nlm.nih.gov/clinvar/variation/VCV000013503.21/; https://www.ncbi.nlm.nih.gov/clinvar/variation/VCV000013505.18/). While it is admittedly rare (total of three heterozygotes and no homozygotes in the gnomAD genomes and a popmax filtering allele frequency of ~ 8 × 10^−6^), the p.Arg457Gln variant was interpreted as a variant of unknown significance (VUS) as there was insufficient evidence to determine its relevance to disease. It has previously been reported as a VUS in a non-neonatal phenotype (Jerath and Shy, 2018; https://www.ncbi.nlm.nih.gov/clinvar/variation/VCV000576988.3), however, those cases were not, to our knowledge in *cis* with T251-P457L or in *trans* with E1136K. One likely pathogenic POLG variant (p.Glu1136Lys) was present on the paternal allele. It has been reported previously in *trans* with the p.Thr251Ile and p.Pro587Leu variants in an individual with infantile hepatocerebral mtDNA depletion syndrome (Morris et al., 1998; Hargreaves et al., 2002; Taanman et al., 2009). The patient also maternally inherited a rare variant in FLNA in a region of the gene not previously known to harbor pathogenic variants. The variant was not seen in the similarly affected sibling. This variant was therefore considered a VUS (see Table 1; Figure 2).

**Table 1:** Variants identified by whole exome sequencing.

Gene	Variant	MAF (gnomAD)^a^	*In silico* damaging predictions^b^	Proband	Mother	Father	Affected Sib	Healthy Sibs	Interpretation
POLG	NM_001126131.1: c.752C>T (p.Thr251Ile)	0.002666	5/10	Het	Het	Ref	Het	Ref	Pathogenic
POLG	NM_001126131.1:c.1760C>T (p.Pro587Leu)	0.002674	10/10	Het	Het	Ref	Het	Ref	Pathogenic
POLG	NM_001126131.1: c.1370G>A (p.Arg457Gln)	0.00001758	5/10	Het	Het	Ref	Het^c^	Ref	VUS
POLG	NM_001126131.1:c.3406G>A (p.Glu1136Lys)	Absent	10/10	Het	Ref	Het	Het	Het	Likely pathogenic
FLNA	NM_001456.3 c.5294C>T (p.Pro1765Leu)	0.00001988	6/10	Het	Het	Ref	Ref	Ref	VUS

Het, heterozygous; Ref, reference (wild-type); VUS, variant of unknown significance; MAF, minor allele frequency.

a Non-Finnish European minor allele frequency except FLNA, which is the global frequency.

b DANN, MutationTaster, FATHMM, FATHMM-MKL, MetaSVM, MetalR, LRT, MutationAssessor, SIFT, and Provean.

c The p.Arg457Gln variant assumed present in the affected sibling due to its presence on the transmitted maternal haplotype.

Thus, the maternal allele had two known pathogenic POLG variants and the paternal allele had one POLG variant considered likely pathogenic. Given these genetic findings with a clinical picture of lactic acidosis, hepatic failure, respiratory failure, and encephalopathy, she was diagnosed with hepatocerebral POLG-related mtDNA depletion syndrome. Genetic testing of the sibling’s frozen fibroblast cells was completed (Table 1) and consistent with this conclusion. The patient developed Klebsiella sepsis with progressive respiratory failure, liver failure, and encephalopathy. Parents withdrew medical intervention at 3 months of age and the patient died.

## Discussion

The diagnosis of a POLG-related mtDNA depletion syndrome is challenging to make in clinical practice. These are extremely uncommon diagnoses, especially in the neonatal population. Symptoms manifest differently across individuals and families. Genetic diagnosis is difficult without a reliable functional assay, so proof that a mutation is pathogenic must be attributed to another case with the same identified variant. Our patient’s symptoms demanded expansion of the differential diagnosis for a cohesive diagnosis. Her respiratory failure was initially attributed to RDS and, at the time of transfer to our facility, bronchopulmonary dysplasia due to prematurity. However, the vast majority of infants born at ~32 weeks GA do not have this degree of respiratory failure, nor do they go on to develop significant chronic lung disease. When her respiratory failure became out of proportion to her GA and she developed progressive liver disease and encephalopathy, we considered other diagnoses. Lactic acidosis provided evidence of energy failure. The family history of a sibling with early mortality from respiratory insufficiency at roughly the same gestational age prompted consideration of an inherited disease contributing to worse than expected outcomes for these moderately premature siblings. Initial genetic testing provided normal results. rES provided multiple variants that, together with her phenotype, allowed us to make the diagnosis of POLG mtDNA depletion syndrome in both her and her deceased sibling.

While it is true that patients with the T251I-P587L haplotype have demonstrated remarkable variation in age of onset when found in *trans* with a range of pathogenic and likely pathogenic variants (Scuderi et al., 2015), in this case there is data on the cooccurrence of this variant with the E1136K variant noted on the paternal allele, which can guide our interpretation of expected impact. Based on POLG pathogenicity prediction server results (Nurminen et al., 2017), this combination of mutations would be expected to have infantile onset. There are three reported patients from Taanman et al. (2009) and Hikmat et al. (2017) who had both E1136K on one allele and T251I-P587L on the other. These patients presented in infancy with hepatocerebral disease including lactic acidosis, jaundice, hepatomegaly, septicemia (specifically in our case, and as reported by Taanman, Klebsiella sepsis), hypotonia, and failure to thrive. Our patient differs from those previously reported in that our patient’s primary presentation was severe respiratory failure, only later evolving into hepatocerebral disease. POLG variants alone have not typically been described as presenting with a lung disease phenotype, though the proportion of reported cases born premature is small.

The respiratory failure in this case was likely multifactorial. RDS was presumably the foundation of her respiratory failure, exacerbated by bronchopulmonary dysplasia and PH. While PH has been described in other mitochondrial disorders, particularly NFU1 deficiency, it is not typically considered a feature of POLG-related mtDNA depletion syndromes. However, PH has also not been considered a key features of DGUOK deficiency, yet Barclay et al. (2005) and Grabhorn et al. (2014) independently describe five such cases in that mtDNA depletion disorder. We can speculate that elevation of the hemi-diaphragm and/or diaphragmatic dysfunction due to energy failure from mtDNA depletion in the setting of increased energy demands of neonatal RDS may have been a contributing factor specific to the POLG mutation. Because of the relative rarity of these disorders and their coincident diagnosis in the setting of prematurity, we arrive at this speculation on the basis of the large body of literature on mitochondrial oxidative stress and diaphragmatic dysfunction in adults, recently summarized by Duan and Bai (2020). Beyond this speculation, there is some data specific to this patient’s POLG variants, which could explain a severe imbalance between demand for POLG activity and function. Debalsi et al. (2017), demonstrated that the T251I-P587L combination causes a synergistic defect leading to poor replication. An explanation of E1136K was provided by Taanman et al. (2009): Glu1136 is a highly conserved amino acid invariant from yeast to man and located in a critical motif of the polymerase domain. This residue, in combination with Asp1135 and Asp890, form a triad of carboxylic acids that are essential for catalysis. A substitution of Glu with a positively charged residue such as Lys would cause a global structural change and eliminate polymerase activity entirely, thus, leaving the only functional POLG allele in our patient to be the poorly functioning T251I-P587L-R457Q allele. Further supporting the view that the T251I-P587L allele in *trans* with a variant that severely disrupts the highly conserved residues 1,134–1,136 will cause severe disease, Darin et al. (2021) biochemically characterized the T251I-P587L allele in *trans* with H1134T as causing severely diminished replication and prominent stalling, particularly in muscle and brain. The exact contribution of the R457Q variant is uncertain. It has previously been reported as a VUS in a non-neonatal phenotype (Jerath and Shy, 2018), however, those cases were not, to our knowledge in *cis* with T251-P457L (or, for that matter, any pathogenic or likely pathogenic POLG variant; indeed, in one of the two cases for which data are available, the variant was not even in *trans* with a pathogenic or likely pathogenic POLG variant). It is possible, though purely speculative, that the co-occurrence of this rare variant with the already well characterized pathogenic T251-P457L further diminished residual function of the maternal allele in the setting of a non-functional paternal allele and against a background of prematurity, increased the liability of the patient and her affected sibling to severe disease and an early respiratory phenotype. The hypothesis of prematurity as an environmental interaction with the POLG variants is not merely speculative. Indeed, given the known metabolic immaturity of the respiratory chain and mitochondrial metabolism more broadly in the setting of prematurity (Sperl et al., 1992; Honzik et al., 2008), it is quite plausible that the patient’s prematurity exacerbated her mtDNA depletion syndrome and led to an earlier presentation than is typical.

Likely pathogenic and pathogenic variants in FLNA have been reported to cause respiratory failure early in life, mostly in the setting of extreme prematurity, but also with periventricular nodular heterotopia (Gerard-Blanluet et al., 2006; De Wit et al., 2011; Masurel-Paulet et al., 2011; Lord et al., 2014), which was not the phenotype noted on imaging. While, we cannot exclude the FLNA variant as the cause of the disproportionate respiratory failure, there is also insufficient evidence to blame this variant for this phenotype, particularly when variants in this region of the gene have not been previously implicated in disease and when there is a similarly affected sibling who did not inherit the FLNA variant, but did inherit all the same POLG variants as our patient.

In addition to the natural history of POLG-related mtDNA depletion disorders described above, increased hepatotoxicity related to valproate use is well described (Stewart et al., 2010). However, while the elevation of transaminases with bosentan in the general population is well described (Valerio and Coghlan, 2009), most such elevations resolve with cessation of the drug within a matter of days. The persistence of transaminase elevation and progression of disease in our patient is notable, but does not establish causality. Given how uncommon POLG variants are and how few neonates receive bosentan, testing the hypothesis that deleterious POLG variants increase the risk for drug induced liver injury with bosentan will be challenging, and likely limited to case control studies for the foreseeable future.

A key limitation in the interpretation of these findings is that we did not obtain quantitation of mtDNA depletion. However, as described above, there is extensive evidence for pathogenicity of each of our patient’s alleles independently as well as multiple described patients with disease from T251I-P587L in *trans* with E1146K. Most importantly, Taanman et al. (2009) quantified liver mtDNA in a neonate with these exact variants at 3% of healthy controls and DNA polymerase gamma activity in fibroblasts as 2.8 pmol dTTP/mg/min compared to a control range of 8.2–19.8 pmol dTTP/mg/min. The presence of the R457Q variant on the T251I-P587L allele may further alter protein function, but it seems improbable that it would rescue functionality to such a point as to rescue the phenotype.

We present a case of a 32-week infant with respiratory failure, PH, liver failure, lactic acidosis, and encephalopathy. Using rES, this patient was diagnosed with POLG-related mtDNA depletion syndrome. This case demonstrates that expanding the differential to uncommon diagnoses is important for complex infants, even in premature neonates whose condition may be explained partly by their GA. It also shows patients with complex neonatal diseases with significant family history may benefit from rES to allow for expedited diagnosis and early termination of the diagnostic odyssey. Indeed, this conclusion is concordant with the findings of both the NSIGHT2 randomized controlled trial (Dimmock et al., 2020) as well as the Australian Acute Care Genomics Study (Australian Genomics Health Alliance Acute Care Flagship et al., 2020). However, those findings had not been published, let alone made it into widespread practice when this patient presented and so this patient initially underwent panel-based genetic testing based on the respiratory failure phenotype then apparent and the causal variants in POLG were not interrogated. Given the protracted course and ultimate outcome, it seems probable that early molecular diagnosis *via* rapid genomic testing could have informed care throughout the admission. Finally, this case provides further evidence that unbiased genomic diagnostics expands the phenotype of rare disease.

## Data Availability Statement

The original contributions presented in the study are included in the article/supplementary material, further inquiries can be directed to the corresponding author.

## Ethics Statement

Written informed consent was obtained from the relevant individual(s) for the publication of any potentially identifiable images or data included in this article. The family gave informed consent for this publication and request of medical records.

## Author Contributions

ADF, KZM, and BPC completed the writing, literature review, and editing of the manuscript. DCK provided the variant analysis, diagram completion, review, and editing of the manuscript. All authors approved the final manuscript as submitted and agree to be accountable for all aspects of the work.

### Conflict of Interest

The authors declare that the research was conducted in the absence of any commercial or financial relationships that could be construed as a potential conflict of interest.
